# Hospital Visitors’ Awareness and Adaptation of Preventive Measures for Middle East Respiratory Syndrome Coronavirus

**DOI:** 10.1017/dmp.2020.435

**Published:** 2020-11-05

**Authors:** Khalid M. Aljarallah, Mohammed O. Alrukban, Yazeed S. Alghamdi, Bandar S. Alanazi, Khaled E. Alturki, Hamad A. Alkhunayfir, Madloul J. Alshammari, Fahad M. Alamri, Hanadi Y. Hamadi

**Affiliations:** 1 College of Applied Medical Sciences, Majmaah University, University Campus, Majmaah City, Riyadh, Saudi Arabia; 2 College of Applied Sciences, Almaarefa University, AlDere’yah, Riyadh, Saudi Arabia; 3 Department of Family and Community Medicine, King Saud University, Riyadh, Saudi Arabia; 4 Department of Health Administration, Brooks College of Health, University of North Florida, Jacksonville, Florida, USA

**Keywords:** awareness, coronavirus knowledge, middle east respiratory syndrome coronavirus, respiratory viral infections

## Abstract

**Objective:**

This study aims to assess the knowledge and awareness, and to identify the practice reflection of knowledge concerning Middle East Respiratory Syndrome (MERS) on hospital visitor’s daily life.

**Methods:**

A cross-sectional study, conducted in 2 tertiary referral hospitals in Riyadh Saudi Arabia, from February 2015 to February 2016. A total random sample of 305 hospital visitors consented to participate. Data were collected through a self-administered questionnaire consisting of questions regarding awareness and practice of measures to prevent the spread of infection.

**Results:**

Study showed that participants have a fair knowledge regarding the cause of MERS (N = 228; 74.8%). Nearly half of them (47%) stated that camels are the source of the spread of MERS. Approximately 70% of the participants preferred both sanitization and wearing facemasks as preventive measures for MERS. However, only 3.95% practiced not eating camel products, such as milk and meat.

**Conclusions:**

Although hospital visitors showed some knowledge and positive awareness in several aspects of MERS awareness, there are weak areas where knowledge and awareness were not up to recommended guidelines. Continued educational programs are needed to improve awareness and knowledge of all the public toward MERS-coronavirus infection. This study may assist in the development of future strategies on preventive measures of the disease.

According to the World Health Organization (WHO), coronaviruses are a threat to the entire world.^1^ The United Nations (UN) health chief has urged global cooperation to tackle novel coronavirus threats by implementing measures such as frequent surveillance, evaluation of awareness, knowledge, and practice of preventive measures.^2^ Middle East respiratory syndrome (MERS) is a viral respiratory disease caused by a novel coronavirus called Middle East respiratory syndrome coronavirus, or MERS-CoV, that was first identified in Saudi Arabia in 2012.^3^ Experts from King Saud University (KSU) and WHO jointly explored MERS virus from nasal swabs of camels and demonstrated that both human and camel had the same genome sequence, showing its direct transmission from camel to human.^4,^^5^

Several other countries, including United Arab Emirates, Kuwait, Qatar, Indonesia, Thailand, United Kingdom, South Korea, China, and the United States, all reported having MERS cases.^6^ Animals, including bats, chimpanzees, and dromedary camels are found to be the natural reservoirs of MERS-CoV.^7-10^ Camels are considered the source of infection transmission to humans. Subsequently, human-to-human transmission of MERS-CoV occurs from patients to health-care workers through droplet infection, or through touching contaminated surfaces. The incubation period of MERS-CoV ranges from 2 to 14 d.^11^ General signs and symptoms consist of rigor, feeling cold along with shivering, migraine, cough, sore throat, difficulty breathing, muscular rheumatism, chest pain, kidney failure, pneumonia, giddiness, nausea and vomiting, dysentery, and stomach pain. It has been reported that abnormal symptoms consisting of slight respiratory infection without pyrexia and diarrhea will occur before the development of pneumonia.

## Significance and Purpose

As preventive measures, WHO has recommended safety guidelines that include vigorous *hand washing* with soap and water for at least 20 s, cleaning hands regularly with disinfectant or alcohol-based hand-sterilizing solution, and warm water may help in preventing disease transmission. Also, disposable gloves should be used in the case of direct contact with an infected person’s body fluids or feces. Covering one’s nose and mouth with a tissue when coughing or sneezing, throwing used tissues in the trash immediately, and then washing hands carefully are all safety measures to reduce the spread of the virus. Recommendations also include frequent disinfecting touched surfaces, such as doorknobs, avoiding face, mouth, and nose touching with unwashed hands, avoiding sharing cups, utensils, or other items with sick people, and using soap and hot water to wash the utensils, towels, bedding, and clothing. Finally, following all infection control measures for at least 10 d even after a patient is completely recovered and is asymptomatic.

In this study, it is hypothesized that hospital visitors have unexplained fear toward MERS. Also, hospital visitors do not use appropriate precautions and prevention methods related to MERS. The objectives of this study were to (1) assess knowledge and awareness of hospital visitors in Riyadh, Saudi Arabia, toward MERS; and (2) identify the practice reflection of knowledge concerning MERS on hospital visitor’s daily life. This research will act as a step forward in improving the awareness of hospital visitors regarding the knowledge, awareness, and practice toward MERS-CoV.

## Methods

### Study Design and Setting

The present study used a cross-sectional study design to assess the knowledge and awareness, and to identify the practice reflection of knowledge concerning MERS on hospital visitor’s daily life. The study period was from February 2015 to February 2016 at 2 hospitals: (1) King Khalid University Hospital and (2) King Saud Medical City (Al Shimisi Hospital).

### Participants

A total of 45 d was randomly selected between December 1, 2015, and February 1, 2016. The 45 d were randomly assigned to each hospital. For each randomly identified day, 4 random 1-h-long time blocks were selected. Two time blocks were restricted to being between 7 am and 3 pm (regular business hours), and the other 2 time blocks were restricted to being between 3 pm and 8 pm (after hours). The start and end times were selected based on hospital policy on the clinic and visiting hours. A total of 120 recruitment time blocks were conducted. Using a nonprobability sampling method, the first 8 participants of each session were invited to participate. A total of 1440 were invited to participate in the survey. A power calculation showed that a sample size of 304 is needed to achieve a confidence level of 95% with 5% margin of error.

### Inclusion Criteria

The study inclusion criteria were adults between the ages of 15 and 59 y, who consented to participate in the study, and are visiting the in-patients/out-patients departments and clinics.

### Ethical Approval

The study protocol (E-18-3652) was approved by the research and ethics committee’s Institutional Review Board at the College of Medicine, King Saud University, and the permission to collect data at 2 hospitals was taken from the hospital authorities. Verbal consent was taken from each participant and recorded. The participants were briefed about the objectives of the study and the potential benefits to the community. Confidentiality was maintained all through the study, and the data were used only for statistical analysis.

### Data Collection Tool

The data were collected through a self-administered questionnaire consisting of 3 sections developed by the research team. The first section included demographic information of the participants. The second section was designed to assess the participant’s knowledge about MERS-CoV, and the third section was about adaptation to the preventive measures of MERS. In addition, participants were asked 11 salient questions in a specific order with an option response of (Yes/No) (Table 1).

**Table 1.** Salient questions asked of participants in specific order, N = 305

Salient Questions (11-Items)	Response
1. MERS-CoV is a viral infection	Yes/No
2. MERS-CoV is associated with a high mortality rate	Yes/No
3. MERS-CoV is transmitted by close contact with an infected person or animal	Yes/No
4. Fever, cough, shortness of breath are the symptoms of MERS-CoV	Yes/No
5. The incubation period of MERS-CoV is 4-8 wk	Yes/No
6. A vaccine against MERS-CoV is available now	
7. Washing hands frequently with soap and water can help in the prevention of the spread of disease	Yes/No
8. Nose and mouth should not be touched with dirty hands	Yes/No
9. MERS-CoV infected surfaces like doorknobs and handles should not be touched	Yes/No
10. Drinking camel milk and eating camel meat should be stopped	Yes/No
11. Contact with patients should be avoided	Yes/No

### Statistical Analysis

The data were analyzed using SPSS 21.0. Means and SDs were reported for continuous variables. Frequencies and percentages were reported for categorical variables. Pearson’s correlation was applied to find an association between continuous variables. A P-value of <0.05 was considered statistically significant.

## Results

A total of 305 Saudi participated in the study, of which 179 (58.7%) were in King Khalid University Hospital and 126 (41.3%) were in King Saud Medical City (Al Shimisi Hospital). The majority of the participants were males 196 (64.3%) as compared with 109 (35.7%) females. The majority of the participants were also living in cities 285 (93.4%). The largest age group of the study was 20- to 29-y-olds followed by 30- to 39-y-olds. Most of the participants visited the hospital as a patient visitors and followers (visitors are those vising inpatient relatives or friends, and followers are those seeking follow-up care) 138 (45.2%), followed by visiting outpatients 127 (41.6%) and inpatients 40 (13.2%). Almost half of the participants were university degree holders, and two-thirds of the participants were working in nonmedical fields (Table 2).

**Table 2.** Demographics of participants, N = 305

Participant Demographics	N (%)
Gender	
Male	196 (64.3%)
Female	109 (35.7%)
Age group (y)	
15–19	30 (9.8%)
20–29	95 (31.1%)
30–39	82 (26.9%)
40–49	59 (19.3%)
50–59	39 (12.8%)
Residence	
City	285 (93.4%)
Village	20 (6.6%)
Hospital	
King Khalid University Hospital	179 (58.7%)
King Saud Medical City (Alshimisi)	126 (41.3%)
Reason for being in hospital	
Inpatient	40 (13.1%)
Outpatient	127 (41.6%)
Patient visitors and followers	138 (45.2%)
Educational level	
Less than high school	37 (12.1%)
High school	116 (38%)
University degree	152 (49.8%)
Job category	
Medically related	31 (10.2%)
Not medically related	195 (63.9%)
Not working	65 (21.3%)
Student	14 (4.6%)

In Table 3, the level of knowledge of participants regarding MERS was presented. Most of the participants 228 (74.8%) knew that MERS is caused by a virus, while 16 (5.2%) and 11 (3.6%) participants thought that it was caused by a bacterial or fungi infection, respectively. Approximately 126 (41%) participants believed that there is a treatment for MERS, while 61 (20%) believed that there is no treatment for MERS. According to participants, the mode of transmission of MERS was through the air or breathing it in 195 (63.9%), by contact with patients 187 (61.3%), and by using personal items of infected persons 153 (50.2%). According to the participants, fever, dyspnea, cough, and nose/throat congestion were the most common symptoms of MERS with percentages of 232 (76.1%), 225 (73.8%), 203 (66.6%), and 150 (49.2%), respectively. The main source of participants’ information about MERS was traditional media 62%, social media 38%, and internet 29%, while MERS information through family, friends, colleagues, books, and brochures was less informative (10%).

**Table 3.** Distribution of participant’s knowledge regarding middle east respiratory syndrome, N = 305

Participant’s Knowledge	N (%)
Kind of germs of MERS	
Virus	228 (74.8%)
Bacteria	16 (5.2%)
Fungi	11 (3.6%)
Not sure	50 (16.4%)
Mode of transmission	
Blood	81 (26.6%)
Breath	195 (63.9%)
Personal items	153 (50.2%)
Contaminated surfaces	103 (33.8%)
Contact with patients	187 (61.3%)
Eating fast food	10 (3.3%)
Camel products	8 (2.6%)
Symptoms	
Dyspnea	225 (73.8%)
Cough	203 (66.6%)
Fever	232 (76.1%)
Nose and throat congestion	150 (49.2%)
Diarrhea	127 (41.6%)
Nausea and vomiting	108 (35.4%)
Syncope	27 (8.9%)
Rash	13 (4.3%)
Bleeding	11 (3.6%)
Epilepsy	3 (1.0%)
Preventive measures	
Wash hands with water and soap	236 (77.4%)
Use hand sanitizer	221 (72.5%)
Wear protective masks	213 (69.8%)
Avoid touch nose and mouth if possible	147 (48.2%)
Not using public utilities	44 (14.4%)
Cover nose and mouth when sneeze or cough	175 (57.4%)
Stop eating meats	12 (3.9%)
Avoid contact with patients	191 (62.6%)
Treatment options	
Yes	126 (41%)
No	61 (20%)
Not sure	118 (39%)

When participant’s awarenesss were examined, nearly half (47%) mentioned that camels are the only source for the spread of MERS, while 20% mentioned that there is no relation between camels and MERS. Less than 12% think that all animals are the source for the MERS spread, while 22% were not sure if there is any relationship between animals and the spread of MERS. Half of the participants (49.5%) preferred to completely avoid any sort of contact with camels and their products, while 8.9% preferred to avoid intake of camel’s meat and milk to protect themselves, and 18.6% said they do not care about it. When participant’s practices were studied, nearly half of them (49%) said they do not worry about visiting a hospital if there is a MERS outbreak, 15% said that they will avoid going to hospitals in the case of an outbreak, and 36% said they will prefer to delay their appointments if there is an outbreak.

Approximately 70% of the participants preferred both sanitization and wearing face masks as preventive measures for MERS. Approximately a quarter (25%) of participants preferred to either use sanitization or wear face masks. Most of the participants (62%) will suggest that individuals with a cough go to the hospitals to seek MERS related diagnostics, while 27% said they will avoid such people completely.

Educational level was associated with participants’ response to the correct answer regarding “kind of germs,” a significant association was observed (P < 0.001) (not shown). However, no significant association was observed between age, job, residency, the reason for being in the hospital, and the correct answer (P > 0.05), respectively. Males believed more that there is an available treatment for MERS as compared to females (P < 0.001). However, no significant association was observed between age, job, and education level with treatment options (P > 0.05, respectively) (see Table 4).

**Table 4.** Distribution of participant’s responses according to availability of treatment, N = 305

	Yes	No	Not Sure	Total	P-Value
	N (%)	N (%)	N (%)		
Age (y)					
15–19	16 (53%)	3 (10%)	11 (37%)	30	
20–29	37 (39%)	23 (24%)	35 (37%)	95	0.423
30–39	37 (45%)	16 (20%)	29 (35%)	82	
40–49	24 (41%)	13 (22%)	22 (37%)	59	
50–59	12 (31%)	6 (15%)	21 (54%)	39	
Gender					
Male	93 (47%)	27 (14%)	76 (39%)	196	<0.001^*^
Female	33 (30%)	34 (31%)	42 (39%)	109	
Job category					
Medically related	13 (42%)	11 (35%)	7 (23%)	31	
Not medically related	85 (44%)	39 (20%)	71 (36%)	195	0.064
Not working	23 (36%)	10 (15%)	32 (49%)	65	
Student	5 (36%)	1 (7%)	8 (57%)	14	
Educational level					0.181
Less than high school	17 (46%)	10 (27%)	10 (27%)	37	
High school	50 (43%)	16 (14%)	50 (43%)	116	
University degree	59 (39%)	35 (23%)	58 (38%)	152	

* Statistical significance P < 0.05.

The association between the spread of MERS and animals shows that male participants said camels are the only source for the spread of the virus as compared to female participants (P < 0.001). Participants in all groups said camels are the only source for the spread of the virus (P = 0.022). Nonmedical field workers said camels are the only source for the spread of the virus (P = 0.002). Participants living in cities mostly said that camels are the only source for the spread of the virus (P = 0.018). However, no significant association was observed between the reason for being in a hospital the and spread of the through animals (P = 0.409).

Statistically, a significant association was observed between participants’ interaction when hearing that there is a relation between camels and MERS (P < 0.001). Almost two-thirds of the participants who believe that all animals are sources of MERS together with (61%) who say that the camel is the only source of MERS prefer to avoid camels and its products completely. However, 51.6% of participants did not care, as they believed there is no relationship between animals and MERS (Table 5).

**Table 5.** Distribution of participants responses according to participant’s interaction when hearing that there is a relation between camels and MERS, N = 305

	Avoid Camel Contact	Avoid Camel Meat and Milk	Don‘t Care	Total	P-Value
Sources of MERS	N (%)	N (%)	N (%)		
					<0.001
All animals are sources for MERS	8 (22.8%)	2 (5.7%)	3 (8.5%)	35	
Camels are the only source for MERS	40 (28%)	8 (5.5%)	8 (5.5%)	143	
No relation between animals and MERS	9 (15%)	5 (8.4%)	31 (51.6%)	60	
Not sure	13 (19.4%)	12 (18%)	15 (22.4%)	67	

When the relationship between “source of information” and participants responded to the correct answer about the “kind of germs” was explored, only social media as a source of information was statistically significant (P = 0.04) with a high number of correct answers about determining the kind of germs. Other sources of information (friends, family, colleagues, the Internet, the media, books/brochures had no association) had no significant association (P > 0.05), respectively.

## Discussion

The present study showed the hospital visitors’ levels of knowledge, awarenesss, and practices about MERS-CoV in Riyadh. To the best of our knowledge, this is the first (KAP) study on hospital visitors to examine their knowledge, awareness, and practice regarding MERS-CoV.^8,9,11^ In the present study, a majority of the respondents gained knowledge about MERS from the media (62%), a study conducted in Jeddah about MERS knowledge showed that 27% of the participants gained knowledge from their College. A study done in Al-Qaseem reported that the Internet was the major source of information (26%) regarding MERS knowledge.^12^ The results of the present study show that participants have a decent amount of knowledge regarding the cause of MERS, (75%) said MERS is caused by a virus.

Our study results are consistent with a study conducted in Riyadh that showed (91.6%) of the participants said that MERS is a viral illness. In our study, most of the participants said that breath (63.9%) is the principal mode of MERS transmission, followed by contact with patients (61.3%). This result when compared with a study conducted in Jeddah,^8^ showed that (64%) of participant thought contact with infected MERS patients is the principal way of disease transmission.

In the present study, the respondents showed a moderate level of hygienic practice with over 70% reported washing hands by water and soap, 69% said to wear protective masks, while nose and mouth should be covered was said by over 60%, as compared to another study that showed high preventive measures toward MERS in public.^11^ Our study result showed a low level of prevention practice. This suggests that precautionary activities for limiting the infection with the MERS virus should be strengthened and encouraged.

In this study, nearly half of the participants (47%) mentioned that camels were the only source for the spread of MERS, while 20% said that there is no relation between animals and spread of MERS, this finding is almost the same as reported by other studies that camels are a source of MERS virus (48.9%)^3^ and can transmit it (48%).^8^ Half of the participants (49.5%) in this study preferred to avoid contact with camels and its products completely, 23% preferred to avoid contact with camels only, while 8.9% participants preferred to avoid camel meat and its milk to protect themselves from MERS and 18.6% participants do not care about it.

In terms of practice, if there is a MERS outbreak, almost half of the respondents (49%) in this study said they will not worry about going to the hospital, while the remaining 51% said either they will avoid going to the hospital or prefer to delay their scheduled appointments. Also, 70% of participants preferred to wear masks and take sanitization precautions when going to the hospital. This means that the participants agreed that, even though hospitals are sterile, they should take care of themselves by using preventive measures.

An important finding in this study was the education of participants, university degree holders had higher knowledge in determining the “kind of germs” as compared to other degree holders. Another important finding in this research is awareness about of availability of treatment for MERS. A high percentage of males said the treatment of MERS is available as compared to females. While female said there is no treatment, which is the truth until now.

Gender, age, and job category were significantly associated with saying that camels are the only source for the spread of the MERS virus. Males slightly said this more than females, almost all age-groups ranging between 15 and 59 y of age said camels are the only source for the spread of MERS, whereas, an interesting finding that nonmedical field workers said that camels are the only source for the spread of MERS. As a comment on these results, what makes the media more credible than other sources is because they rely on teamwork and verify accuracy about providing information. However, it is a good sign that social media has begun to provide true information, especially regarding health aspects.

### Limitations

There are several studies on awareness of MERS-CoV among hospital-associated personnel, such as health-care workers, dental students, nurses, and physicians, but the present study is the first to evaluate the practice of preventive measures against MERS-CoV by the general public.

The present study was conducted in 1 city, and the sample size was low considering Riyadh is a cosmopolitan city. The results of the present study cannot be generalized to all of the Saudi population. However, the strength of the study is that it is done on the general public and can provide a magnitude of awareness, and practice of measures to prevent MERS virus infection in the public community and to set educational programs accordingly.

## Conclusions

Although hospital visitors showed a great deal of knowledge and positive awareness in several aspects of MERS-CoV awareness, there are weak areas where knowledge and awareness are not as desired, for instance disagreement to avoid milk and eating the meat of a camel. To limit the spread of MERS-CoV in Saudi Arabia, continued educational programs are needed, to improve awareness knowledge and awareness of the whole public toward MERS-CoV infection. The study can set a paradigm to make future strategy on preventive measures of the disease and similar highly infectious viral diseases. More efforts, vigilance, and surveillance are still needed to raise awareness and to limit and prevent life-threatening MERS-CoV infection. Future research needs to expand on this study by examining the association of hospital visitor knowledge and awareness of coronavirus disease 2019 (COVID-19) preventive measures and its impact on hospital access and safety.
